# Strategies to address conspiracy beliefs and misinformation on COVID-19 in South Africa: A narrative literature review

**DOI:** 10.4102/hsag.v27i0.1851

**Published:** 2022-11-08

**Authors:** Nokwanda E. Bam

**Affiliations:** 1 School of Nursing, Faculty of Health Sciences, North-West University, Mahikeng, South Africa

**Keywords:** strategies, address, COVID-19, vaccines, conspiracy, misinformation

## Abstract

Conspiracy theories and misinformation have been explored extensively however, strategies to minimise their impact in the context of coronavirus disease 2019 (COVID-19) vaccines are limited. This study aimed to explore strategies that can be used to reduce the negative effects of conspiracies and misinformation about SARS-CoV-2. This review was carried out based on accessed literature on beliefs in misinformation about the COVID-19 pandemic. A comprehensive search of databases, such as Google Scholar, EBSCOhost and African Journals between 2019 and 2022 yielded qualitative and quantitative studies. Two themes emerged, namely underlying motives for conspiracy theories and belief in misinformation about the pandemic and ways to overcome them. The latter included: (1) strengthening critical scanning of information, (2) critical review to address misinformation and (3) establishing approaches for managing conspiracy theories. A proposal is made to address conspiracy beliefs about COVID-19 infection.

**Contribution:**

This is believed to be the first review that describes strategies to mitigate belief in conspiracies and misinformation to promote vaccination.

## Background

This study explores and describes strategies to reduce the negative effects of conspiracy theories about the coronavirus disease 2019 (COVID-19) vaccines and related protocols, in order to increase their acceptability and uptake and so reduce the spread of COVID-19 in South Africa (SA). Beliefs in conspiracy theories and misinformation have existed throughout human history, fuelled by rapid and uncertain societal changes (Douglas et al. 2019:3). They can have serious consequences (Van Mulukom et al. 2022:2). From the beginning of the COVID-19 pandemic, messages circulated on social media stating that the disease was intentionally introduced by the Chinese to start a war on America (or vice versa) (Douglas 2020:270). Some reporters affirmed that some people believe that 5G mobile phone networks were associated with the spread of COVID-19 through the radiation they transmit, leading to attacks on cell towers in some parts of the world, including Europe (De Coninck et al. 2021:2). Hornsey and Fielding (2017:462) asserted that people who believe in conspiracies attest to the existence of groups of powerful people with malicious intent behind particular events and, coincidentally, reject the contradictory scientific evidence. In the context of COVID-19, the effects of conspiracies are reported to have negative outcomes on vaccination against coronavirus and related protocols (Douglas et al. 2019:18; Pummerer et al. 2020:2; Soteri et al. 2021:684).

The African continent has not been spared the destructive effects of COVID-19. The Africa Centre for Disease Control and Prevention (Africa CDC 2020:n.p.) recorded that some countries, such as Burundi, the Democratic Republic of Congo, Sudan, Algeria and Egypt, have reported case fatality rates higher than the global case fatality rate of 6%. As the coronavirus is highly contagious, with deadly health outcomes, governments all over the world have issued safety measures to control the spread of severe acute respiratory syndrome coronavirus 2 (SARS‑CoV‑2), which is the viral agent responsible (Van Mulukom et al. 2022:3). However, there is widespread lack of adherence to safety protocols (Pummerer et al. 2020:1). Furthermore, trust and confidence in the efficacy of vaccines is compromised by people who refuse to accept their value (Bokemper et al. 2021:825; Douglas 2021:3; Imhoff & Lamberty 2020:1114). For example, in May 2021, the South African Government Communication Department (2021:n.p.) published guidelines to tackle misinformation related to COVID-19 in the hope that two-thirds of the population would get vaccinated to achieve herd immunity. Some of the issues that raise distrust and rejection of vaccines include the belief that COVID-19 vaccines will change a person’s deoxyribonucleic acid (DNA), that they contain some microchip device that will be used to track people’s whereabouts and that they are a way for the oppressors in the past to control people again – all of which are refuted by the facts, as further elaborated on by the SA Communication Department.

Previous studies have focused on understanding beliefs in conspiracies and misinformation (Douglas et al. 2019:3; Van Mulukom et al. 2022:1) and their impact on COVID-19 vaccines and related protocols (Dyrendal & Jolley 2020:2; Heiss, Gell & Rothlingsch 2021:3). However, there is limited information on efforts to manage these beliefs (De Coninck et al. 2021:11; Douglas 2020:274), especially for the purpose of overcoming resistance to vaccination, which has now become an urgent matter.

In South Africa, as of 06 October 2021, out of a total adult population (18 years and older) of nearly 40 million, only 9.53 m individuals were fully vaccinated – that is, they had received one dose of the Johnson & Johnson or two doses of the Pfizer vaccines – representing just 24% of the adult population. The reluctance of so many South Africans to get vaccinated highlights the core of the problem: that more studies need to understand the challenges to achieve general vaccination (De Coninck et al. 2021:1; Douglas 2021:1). Failure to address COVID-19-related conspiracies and misinformation, which can be promoted by high-profile individuals including politicians and scientists (Bierwiaczonek, Kunst & Pich 2020:1271; Bokemper et al. 2021:825; Duplaga 2020:1), will delay eradication of COVID-19 pandemic.

Eysenbach (2020:n.p.) coined the concept of ‘infodemiology’ in the context of health and misinformation. Furthermore, unpacking the destructive nature of ‘infodemics’, the Africa Infodemic Response Alliance (AIRA) (2020:n.p.) averred that they make susceptible individuals confused and lead to mistrust in public interventions, eventually causing death if not properly managed (Mbunge 2020:1810). Given that infodemics tend to spread widely within a short time, the World Health Organization (WHO) attempted to define the term as the outbreak of context-based reliable and unreliable information during epidemics (Tangcharoensathien et al. 2020:n.p.). It is on these grounds that the author, having been exposed to conspiracy theories promoted by people in social networks about COVID-19 vaccines, embarked on this study to seek ways of addressing them in the context of South Africa. It is hoped that the outcomes of this investigation will increase vaccine acceptability and adherence to related protocols, in order to achieve the required herd immunity to eradicate COVID-19 (AIRA 2020; Bertin, Nera & Delouvée 2020:8; Eysenbach 2020:n.p.).

## Author’s motivation

The author has personally experienced both the devastating effects of COVID-19 infection and the corresponding conspiracy theories and misinformation about its source and treatment. In the first week of June 2020, the author experienced flu-like symptoms such as abdominal pains, weakness of joints and loss of taste. By that time the symptoms of COVID-19 were known, and relevant statistics were being received daily via mobile phones, the radio and websites, so she went to get tested. When the results came back within a week, the symptoms had subsided except for persistent headaches. However, checking the facts on COVID-19 from the South African government website to deal with personal sense of shame, self-blame and misinformation to cope with anxiety and fears was of great help in challenging her thinking. Furthermore, recovery from COVID-19 and lessons drawn from exposure to misinformation about the disease has given her an opportunity to be an ambassador of spreading science-based information on COVID-19. But the experience has left an unanswered question. In addressing your concern on the statement below, how about if we add,’ which is expressed by the author as follows:

What about the people who do not have resources such as access to the Internet or basic forms of communication? How do they access scientific information to make informed decisions and protect or heal themselves with the relevant communication?

The author has witnessed with concern her students’ reluctance to accept COVID-19 vaccines during a 3-month community project campaign between July 2021 and September 2021, hosted by the institution where the study was conducted. One of the concerns the students raised is the fear of infertility and death caused by the vaccines.

Noting some challenges, including poor health literacy, that may prevent the African continent from mounting an effective response to misinformation about COVID-19, Lucero-Prisno, Adebisi and Lin (2020:1) advocated for approaches that may counteract the problem. For example, critical thinking skills can be taught to check for facts and logic on sources of information as opposed to passive acceptance of convincing but false news (Dyrendal & Jolley 2020:7), such as ‘the side effects of the vaccine will be worse than having COVID-19’, and the ‘vaccine causes paralysis’. These beliefs impacted the author so negatively that there were notably delays in acceptance of COVID-19 vaccination to the last week of the 3 months of the campaign. Furthermore, there was a sense of curiosity to watch and see what would happen to colleagues who were vaccinated, whether they would experience any untoward symptoms or even deadly outcomes from the vaccines before acceptance of vaccines. Therefore, personal decisions to embark on the following steps in any information that came through from social media or verbal sources were made:

- Check the credibility of information against some research websites, for example, PubMed, Google Scholar.
- Delete it immediately to avoid sharing unfounded information.
- Warn others about the false message by passing on the better-founded facts, starting with those from the social networks who may have forwarded it.

Falade (2019) argued from the African perspective that there is close a relationship between religious and political authorities and the uptake in scientific information. The author of this review is in full agreement with this view as her decision to accept COVID-19 vaccines was motivated by religious authorities in high ranking within the church and the president of SA, Mr Ramaphosa, who led by example and took the first steps to get vaccinated. Consequently, the role-modelling actions displayed by the leaders strengthened the personal choice to let go of conspiracy and misinformation beliefs.

Thus, the author seeks answers to the question: what are the strategies available in literature that could be used to address conspiracy beliefs and misinformation?

## Objectives

This study aimed to explore and describe strategies that can be used to reduce the negative effects of conspiracies and misinformation about COVID-19 in order to increase vaccination intentions.

## Design and methods

**FIGURE 1:**
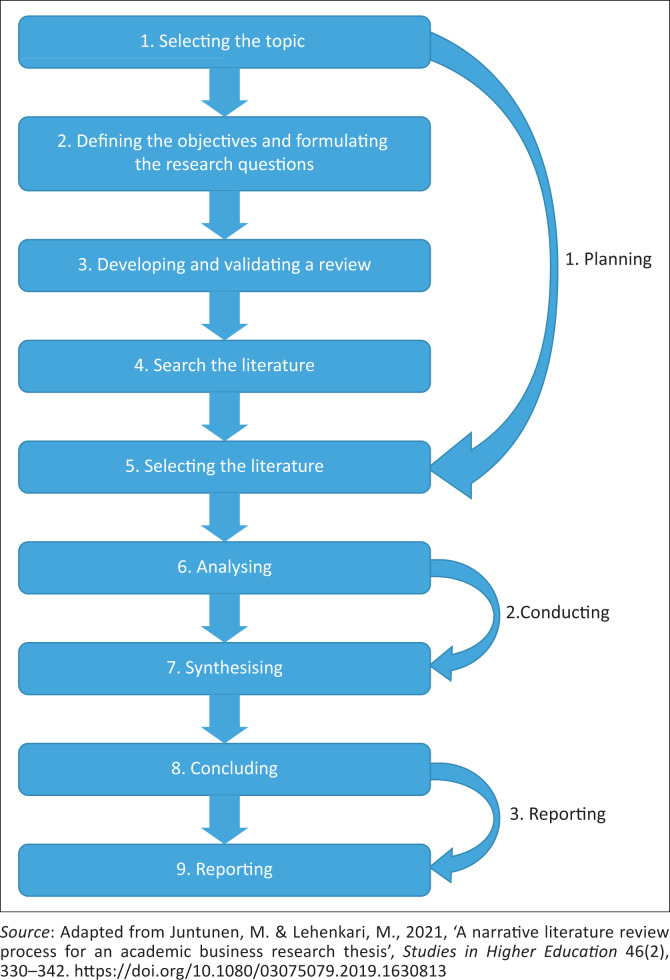
Narrative literature review steps.

This study adopted the narrative literature review approach to address the author’s concern about COVID-19- related conspiracy beliefs and misinformation. It involved comprehensive and critical analysis of the literature to locate the study within the body of knowledge in a particular focus on identifying gaps in the general public’s knowledge of the pandemic (LoBiondo-Wood & Haber 2014:21). The literature search was conducted using Google Scholar, the WHO website, the South African Department of Health website, the Africa CDC and North-West University Library through the e-Link Catalogue, seeking journal and conference publications issued between 2019 and 2022. The search terms used are listed in Table 1. Juntunen and Lehenkari (2021:332) proposed three phases for the purpose, including planning, conducting and reporting, as illustrated in Figure 1.

**TABLE 1:** Elements of the searched literature.

Visited-databases and websites	Terminology used	Criteria of inclusion	Criteria of exclusion
Google Scholar EBSCOhost WHO Africa CDC Department of Health (guidelines)	COVID-19 conspiracy COVID-19 misinformation Conspiracy strategies	English articles that address strategies used on COVID-19 conspiracy theories and misinformation, from the years 2019–2022	Newspaper articles Reports Websites that were not listed in the review

COVID-19, coronavirus disease 2019; WHO, World Health Organization; CDC, centers for disease control and prevention.

### Ethical considerations

The study was conducted ethically from concept formation stage of the review, execution and write-up of the manuscript findings.

## Results

**FIGURE 2:**
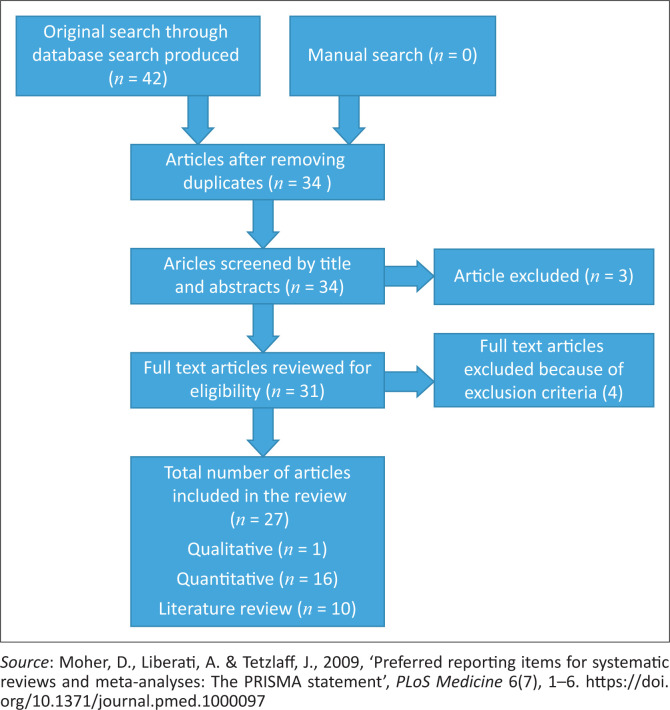
The PRISM flowchart of the systematic review procedure with screening, inclusion and exclusion criteria.

The search strategy results are provided in Figure 2 and accessed studies in Table 2. All articles were evaluated for rigour based on purpose and objectives, design or strategies used, findings and implications or conclusions and quality rank appraisal in terms of A, B and C relating to high-quality grade, good-quality grade and low-quality grade, respectively.

**TABLE 2:** Accessed literature for review.

Author(s) and year	Purpose	Design or strategies and sampling	Rigor or trustworthiness
Adebisi, Rabe and Lucero-Prisno (2021)	To catalogue risk communication and community engagement strategies for COVID-19	Narrative review in 13 countries	Purpose was clearly stated Strategy utilised was appropriate Findings were accurate and fair Conclusion was mentioned. **Appraisal of quality-grade is HQ = A**
Banai, Banai and Miklousic (2021)	To assess the relationship between conspiracy beliefs and adherence to COVID-19 medical guidelines	1882 adults from Croatia, online study	Purpose was clearly stated Strategy utilised was appropriate Findings were accurate and fair Conclusion was mentioned. **Appraisal of quality-grade is HQ = A**
Bertin et al. (2020)	To examine the relationship between COVID-19 conspiracy beliefs, vaccine attitudes and intention to be vaccinated	Two cross-sectional studies: Exploratory on *N* = 409 Extended in a preregistered study (*N* = 396)	Purpose was clearly stated Strategy utilised was appropriate Findings were accurate and fair Conclusion was mentioned. **Appraisal of quality-grade is HQ = A**
Bierwiaczonek et al. (2020)	To determine the relationship between conspiracy and compliance to COVID-19 social distance	Longitudinal survey at five time points in US (*N* = 403)	Purpose was clearly stated Strategy utilised was appropriate Findings were accurate and fair Conclusion was mentioned. **Appraisal of quality-grade is HQ = A**
Carey et al. (2021)	To investigate the effectiveness of interventions aimed at combating false information about the Zika epidemic	Nationally registered face-to-face survey in Brazil Preregistered survey experiments on Brazilian adults	Purpose was clearly stated Strategy utilised was appropriate Findings were accurate and fair Conclusion was mentioned. **Appraisal of quality-grade is HQ = A**
Douglas et al. (2019)	To understand and describe conspiracy theories	Review of multidisciplinary articles	Purpose was clearly stated Strategy utilised was appropriate Findings were accurate and fair Conclusion was mentioned. **Appraisal of quality-grade is HQ = A**
Douglas (2020)	To explore the potential dangers of COVID-19 conspiracy theories	Literature review	Purpose was clearly stated Strategy utilised was appropriate Findings were accurate and fair Conclusion was mentioned. **Appraisal of quality-grade is HQ = A**
Douglas (2021)	To describe the challenges of dealing with negative conspiracy theories	Literature review	Purpose was clearly stated Strategy utilised was appropriate Findings were accurate and fair Conclusion was mentioned. **Appraisal of quality-grade is HQ = A**
Duplaga (2020)	To assess prevalence of conspiracy beliefs in conspiracy theories related to COVID-19	Online survey, *N* = 1002, Polish	Purpose was clearly stated Strategy utilised was appropriate Findings were accurate and fair Conclusion was mentioned. **Appraisal of quality-grade is HQ = A**
Dyrendal and Jolley (2020)	To explore and describe problems and possible solutions of conspiracy theory in the classroom	Surveys to interview teachers and students	Purpose was clearly stated Strategy utilised was appropriate Findings were accurate and fair Conclusion was mentioned. **Appraisal of quality-grade is HQ = A**
Eysenbach (2020)	To describe ways of fighting COVID-19 infodemics	Commentary on how to manage COVID-19 infodemics	Purpose was clearly stated Strategy utilised was appropriate Findings were accurate and fair Conclusion was mentioned. **Appraisal of quality-grade is HQ = A**
Featherstone, Bell and Ruitz (2019)	To assess how the health information sources people, rely upon and their political ideologies are associated with acceptance of vaccine conspiracies	Online survey (*N* = 599) on Amazon’s Mechanical Turk crowdsource platform	Purpose was clearly stated Strategy utilised was appropriate Findings were accurate and fair Conclusion was mentioned. **Appraisal of quality-grade is HQ = A**
Hawley (2019)	To explore parallels of conspiracy theories and impostor syndrome and distrust	Literature review	Purpose was clearly stated Strategy utilised was appropriate Findings were accurate and fair Conclusion was mentioned. **Appraisal of quality-grade is HQ =A**
Guan, Liu and Yuan (2021)	To evaluate the effectiveness of five approaches to reducing conspiratorial belief	*N* = 607 experimental study	Purpose was clearly stated Strategy utilised was appropriate Findings were accurate and fair Conclusion was mentioned. **Appraisal of quality-grade is HQ = A**
Grimes (2021)	To posit a continuum for acceptance of medico-scientific consensus in the context of COVID-19 conspiracy	Literature review	Purpose was clearly stated Strategy utilised was appropriate Findings were accurate and fair Conclusion was mentioned. **Appraisal of quality-grade is HQ = A**
Hammad et al. (2021)	To measure misconceptions towards coronavirus in the Jordanian population	2544 participants from the Jordanian online survey	Purpose was clearly stated Strategy utilised was appropriate Findings were accurate and fair Conclusion was mentioned. **Appraisal of quality-grade is HQ = A**
Heiss et al. (2021)	To investigate how threat perceptions, relate to learning in conspiracy claims about COVID-19	Questionnaire	Purpose was clearly stated Strategy utilised was appropriate Findings were accurate and fair Conclusion was mentioned. **Appraisal of quality-grade is HQ = A**
Imhoff and Lamberty (2020)	To examine relationship between conspiracy belief and COVID-19	Survey in three studies (*N* = 220; *N* = 288; *N* = 298)	Purpose was clearly stated Strategy utilised was appropriate Findings were accurate and fair Conclusion was mentioned. **Appraisal of quality-grade is HQ = A**
Lucero-Prisno et al. (2020)	To provide critical commentary on the current efforts against COVID-19 and challenges in African countries	Commentary	Purpose was clearly stated Strategy utilised was appropriate Findings were accurate and fair Conclusion was mentioned. **Appraisal of quality-grade is HQ = A**
Mbunge (2020)	To conduct literature review on COVID-19 reports, policies	Literature review	Purpose was clearly stated Strategy utilised was appropriate Findings were accurate and fair Conclusion was mentioned. **Appraisal of quality-grade is HQ = A**
Mohammad and Motlaq (2021)	To explore people’s perceptions of COVID-19 through their comments on social media	Qualitative content analysis of over 10 COVID-19 posts on Facebook, reactions from 60 to 701.	Purpose was clearly stated Strategy utilised was appropriate Findings were accurate and fair Conclusion was mentioned. **Appraisal of quality-grade is HQ = A**
Pummerer et al. (2020)	To investigate the gaps on COVID-19 conspiracy theory	National random sample survey, an experiment, and a longitudinal study (1213)	Purpose was clearly stated Strategy utilised was appropriate Findings were accurate and fair Conclusion was mentioned. **Appraisal of quality-grade is HQ = A**
Romer and Jamieson (2020)	To assess acceptance of conspiracy theory circulating in main stream social media	Survey (*N* = 1050); follow-up (*N* = 840)	Purpose was clearly stated Strategy utilised was appropriate Findings were accurate and fair Conclusion was mentioned. **Appraisal of quality-grade is HQ = A**
Sallam et al. (2020)	To evaluate mutual effects of belief that the pandemic was the result of a conspiracy on knowledge and anxiety levels	Electronic based surveys (*N* = 1540), Jordan	Purpose was clearly stated Strategy utilised was appropriate Findings were accurate and fair Conclusion was mentioned. **Appraisal of quality-grade is HQ = A**
Soteri et al. (2021)	To investigate if people’s response to the official recommendations during the COVID-19 pandemic is associated with conspiracy beliefs related to COVID-19	Online survey of 1325 Finnish adults	Purpose was clearly stated Strategy utilised was appropriate Findings were accurate and fair Conclusion was mentioned. **Appraisal of quality-grade is HQ = A**
Tangcharoensathien et al. (2020)	To respond to infodemics related to COVID-19	Online crowdsourcing of multidisciplinary professionals	Purpose was clearly stated Strategy utilised was appropriate Findings were accurate and fair Conclusion was mentioned. **Appraisal of quality-grade is HQ = A**
Van Mulukom et al. (2022)	To pay special attention to cross-national differences, the variety of COVID-19 protective behaviours and different COVID-19 conspiracy theories	Literature review from 85 cross-national articles	Purpose was clearly stated Strategy utilised was appropriate Findings were accurate and fair Conclusion was mentioned. **Appraisal of quality-grade is HQ = A**

COVID-19, coronavirus disease 2019.

Key: HQ = A: high-ranked quality grade; GQ = B: good quality grade; LQ = C: low-ranked quality grade.

## Themes in the literature

Twenty-seven articles relevant to this review were identified, read and synthesised to draw the conclusions relevant to the research question (Table 2). The results of the review yielded two themes, including reasons underlying conspiracy beliefs and misinformation on COVID-19 and their communication strategies. The latter are discussed here under three subthemes.

**Reasons for beliefs in conspiracy theories and misinformation on COVID-19**: These beliefs vary in scope and magnitude but at core represent a psychological need for knowledge and clarity (Douglas et al. 2021:1; Dyrendal & Jolley 2020:1). They usually spread faster when there is lack of information in times of uncertainty as a way to satisfy curiosity and clear uncertainties (Douglas 2019:7, 2020:271). A person who holds conspiracy beliefs has an intrinsic desire to make sense of experiences and circumstances to gain control and eliminate the perceived threat (Hawley 2019:977; Heiss et al. 2021) by believing in alternative information to deal with ambiguity (De Coninck et al. 2021:9). A study by Mohammad and Motlaq (2021:68) on Facebook on a COVID-19 post reported that most people with conspiracy beliefs reacted with negative and pessimistic comments compared with those with positive views and belief in scientific information. Thus, increased feelings of anxiety and depression are associated with higher conspiracy beliefs and misinformation on the pandemic (De Coninck et al. 2021:1). Thus, there is a need to address these beliefs in order to escalate public immunisations (Bertin et al. 2020:8) and should include communication on conspiracies (Douglas 2019:22) to enhance the response to this health crisis (Douglas 2021:4).

## Communication strategies to mitigate beliefs in conspiracy theories and misinformation

Three themes emerged from the literature:

1. Strengthen the review, scanning and verification of information

The WHO’s Information Network for Epidemics (EPI-WIN) was established in December 2019 following the coronavirus outbreak in Wuhan, China, as a resource to give updates and respond to questions related to the epidemic (WHO 2021:n.p.). It handles information, rejects misinformation and addresses COVID-19 infodemics through networks with organisations around the world to provide scientific information. In the report on the COVID-19 response released in February 2021, the WHO Regional Office for Africa acknowledges the supportive role the organisation played in coordinating the global, African and national responses to the pandemic to mitigate risk communication (WHO Africa Region 2020:5). Consequently, COVID-19 websites that provide accurate, up-to-date information on statistics, vaccines and related protocols exist in most countries. Romer and Jamieson (2020:1) added that it is critical that health authorities provide citizens with specific messages via unbiased media.

2. Critical interpretation and explanation of what is known and to address misinformation

The WHO regional office in Africa established risk communication in 47 member states, so that people at risk are able to make informed decisions and alleviate obvious threats, such as COVID-19 infection, to protect themselves (WHO Regional Office for Africa 2020:30). This report explains that it is inadequate simply to provide information on the causes and transmission of coronavirus but that it is imperative to adopt individualised approaches to support families and communities with key messages that encourage the changes we want to see. Supportive strategies, for example, include paying attention to fears and uncertainties, allowing people to express concerns and engaging communities to address risks based on their contexts (Adebisi et al. 2021:139; WHO Regional Office for Africa 2020:30). Furthermore, Hawley (2019:977) warned that whilst it is not easy to change people’s thinking patterns, ensuring dialogue, giving individualised support and dealing with social triggers of conspiracy beliefs and misinformation would go a long way in solving the problem.

3. Analysis of information and critiques of factors affecting behaviour and public health interventions

Dealing with conspiracy theories and misinformation is difficult; hence, strategies to mitigate their effects need to be sought (Bertin et al. 2020:8; Douglas 2021:4; Douglas et al. 2019:21). Amongst these are the following:

- **Scientific information**: Guan et al. (2021:69) expanded on this by focusing on the education of citizens, especially the media, so that they are equipped with the skills to access, analyse and critique media-related issues such as information that may trigger fears in listeners and readers. This requires the cooperation of the authorities, particularly politicians and journalists, who often lead people with different belief systems; thus, if they lead by example, people may be influenced positively to accept vaccines without misinformation (Romer & Jamieson 2020:7), since conspiracy beliefs can sometimes be caused by misinformation and mistrust in authority figures and media (Van Mulukom et al. 2022:24). Furthermore, there is a need to focus on positive information to reach citizens young and old, as opposed to negative information that focuses on the number of people who got infected with COVID-19 or died versus those who recovered (Hammad et al. 2021:1669).
- **Inoculation**: In a medical setting, a person may be given a live weakened vaccine to initiate a mild antibody-antigen response; thus, a person may be protected from severe illness after exposure to a virus, as not all COVID-19 vaccines were live attenuated. Douglas et al. (2019:23) and Douglas (2020:272) asserted that if people are ‘inoculated’ with factual information before they actually encounter it, this knowledge may reduce the effect of subsequent misinformation. Dyrendal and Jolley (2020:6) described this approach as involving three steps, namely (1) a person is warned about the upcoming threat; (2) they are given a pre-emptive refutation; and (3) they are then presented with misinformation to sensitise them before actual exposure to misinformation.
- **Intervention by experts**: Use of experts in the subject area, such as physicians, virologists and immunologists, may help the public to gain confidence and trust in the medical fraternity and comply with the protocols they advocate (Grimes 2021:2). In contrast, if a recognised member of a conspiracy forum argues against conspiracy theories, it may be better received than when the arguments come from scientists or politicians (Douglas 2020). Interventions that seek to coordinate and disseminate effective information build partnerships that include governments, the media, health professionals, law enforcement officers, community leaders, academics and others in order to share best practices and resources (Eysenbach 2020:n.p.). This author further advocates for communication channels that are adapted locally to transmit the WHO’s recommended content on COVID-19 to reach all individuals within a society via affordable platforms such as word of mouth, social media, radio, TV, news or influential people to dispel conspiracies with trusted sources. Failure to address misinformation will promote the spreading of conspiracy theories (Hawley 2019:979) and undermine attempts to eradicate the coronavirus through vaccines, physical distancing and hygiene measures.

## Discussion

The fundamental reason underlying beliefs in conspiracies is described in this review as a psychological need for knowledge (Dyrendal & Jolley 2020:1). Other explanations used to account for them include an attempt to seek clarity (Douglas 2021:1) and a drive to gain insights into one’s experiences during a crisis (Hawley 2019:977; Heiss et al. 2021). In the absence of accepted information, alternative sources are sought, including conspiracy theories and misinformation (De Coninck et al. 2021:7). Carneh and Schrieder (2021:2) challenged this attitude of passive, uncritical acceptance of knowledge with a scientific attitude that seeks to ask the right questions, motivated by basic research to find the right answers, as an attitude to be adopted. Romer and Jamieson (2020:1) acknowledged that in the context of COVID-19, conflicting messages from public health officials, politicians and the media have made it difficult for the public to reach consensus and adopt effective preventive behaviours, especially during the crisis stage when COVID-19 was a new phenomenon for the entire world. Thus, it is important that governments take initiative and provide leadership to guide citizens with accurate, up-to-date information and ease anxieties and panic amongst its citizens (De Coninck et al. 2021:1). It is also observed that as scientific information during COVID-19 has kept changing remarkably – for example, as new variants of the virus appeared – the efficacy of vaccines also changed; thus, the instability of information may perpetuate distrust of scientific evidence on vaccines. However, the communication strategies proposed in this article could be used to address the need for knowledge with evidence-based information to counteract conspiracy theories and belief in misinformation to reduce the negative effects of these beliefs on the COVID-19 pandemic and escalate vaccine acceptability and compliance with the protocols aimed at eradicating coronavirus.

### Limitations

This study has its limitations. Firstly, it focused on strategies to mitigate misinformation about COVID-19 pandemic only; those used during other pandemics, such as SARS, might also be relevant today but are not referred to here. Secondly, scanty information was retrieved that could be included in the review, which is an indication that more research is needed in this area related to COVID-19 misinformation. Lastly, research in other languages apart from English could have positively contributed to the strategies in the current review that could mitigate the two beliefs in the context of COVID-19 pandemic.

### Recommendations

This review has identified major themes that are critical in understanding and mitigating COVID-19 conspiracy theories and misinformation, namely the underlying motives for these beliefs and communication strategies that could be used to reduce their negative effects and to increase the public’s desire to get vaccinated and so eradicate COVID-19.

### Teaching and learning

I recommend that a culture of science-based information be established in education and learning institutions from early grades to tertiary levels to inculcate the art of searching, analysing and checking facts on all information that learners encounter. In this way, learners would grow into being critical seekers, whether they are in online spaces or in interactions with one another, so that conspiracy and misinformation beliefs can be confronted and named for what they are. Thus, if they are inoculated with facts from an early age, they could recognise conspiracy and misinformation beliefs.

### Future research

There is a need for more studies on the strategies that could mitigate conspiracy and misinformation beliefs in order to eradicate SARS-CoV-2. Future research could be conducted using different methodologies such as quantitative, qualitative, mixed methods and multiple methods. There is also a need to examine the effects of these communication strategies on COVID-19 vaccine acceptability and uptake.

### Practice

Health facilities, media personnel and the general public need to be conscientised on interventions that could mitigate conspiracy beliefs or fight misinformation on SARS-CoV-2 with scientific information, taking into cognisance cultural backgrounds, individual situations and their diverse context.

## Conclusion

To the author’s knowledge, this is the first report on strategies to minimise the negative effects of conspiracy theories and misinformation about COVID-19 that undermine vaccine acceptance and protocols. Further research should use multiple approaches to test these communication strategies, specifically in the South African context. The literature shows that conspiracies and misinformation can prevail during pandemics and, although they are hard to control, they can be managed.
